# Effects of endogenous inflammation signals elicited by nerve growth factor, interferon-γ, and interleukin-4 on peripheral nerve regeneration

**DOI:** 10.1186/s13036-019-0216-x

**Published:** 2019-11-13

**Authors:** Chien-Fu Liao, Chung-Chia Chen, Yu-Wen Lu, Chun-Hsu Yao, Jia-Horng Lin, Tzong-Der Way, Tse-Yen Yang, Yueh-Sheng Chen

**Affiliations:** 1 Department of Biological Science and Technology, China Medical University, Taichung, Taiwan; 2 Linsen Chinese Medicine and Kunming Branch, Taipei City Hospital, Taipei, Taiwan; 3 Department of Chinese Medicine, Show Chwan Memorial Hospital, Chunaghua, Taiwan; 4 Department of Chinese Medicine, Chang Bing Show Chwan Memorial Hospital, Changhua, Taiwan; 5 Lab of Biomaterials, School of Chinese Medicine, China Medical University , Taichung, Taiwan; 6 Biomaterials Translational Research Center, China Medical University Hospital, Taichung, Taiwan; 7 Department of Bioinformatics and Medical Engineering, Asia University, Taichung, Taiwan; 8 Department of Fiber and Composite Materials, Feng Chia University, Taichung, Taiwan; 9 Department of Medical Research, China Medical University Hospital, Taichung, Taiwan; 10 Center for General Education & Master Program of Digital Health Innovation, China Medical University, Taichung, Taiwan; 11 College of Humanities and Sciences, China Medical University, Taichung, Taiwan

**Keywords:** Inflammation signals, NGF, IFN-γ, IL-4, Peripheral nerve regeneration

## Abstract

**Background:**

Large gap healing is a difficult issue in the recovery of peripheral nerve injury. The present study provides in vivo trials of silicone rubber chambers filled with collagen containing IFN-γ or IL-4 to bridge a 15 mm sciatic nerve defect in rats. Fillings of NGF and normal saline were used as the positive and negative controls. Neuronal electrophysiology, neuronal connectivity, macrophage infiltration, location and expression levels of calcitonin gene-related peptide and histology of the regenerated nerves were evaluated.

**Results:**

At the end of 6 weeks, animals from the groups of NGF and IL-4 had dramatic higher rates of successful regeneration (100 and 80%) across the wide gap as compared to the groups of IFN-γ and saline controls (30 and 40%). In addition, the NGF group had significantly higher NCV and shorter latency compared to IFN-γ group (*P* < 0.05). The IL-4 group recruited significantly more macrophages in the nerves as compared to the saline controls and the NGF-treated animals (*P* < 0.05).

**Conclusions:**

The current study demonstrated that NGF and IL-4 show potential growth-promoting capability for peripheral nerve regeneration. These fillings in the bridging conduits may modulate local inflammatory conditions affecting recovery of the nerves.

## Background

Peripheral nerve regeneration represents a series of highly specialized processes of cellular and molecular events [1]. The techniques involving use of artificial tubes to bridge a severed nerve provide a means for studying these regenerative processes directly [2–5]. Several kinds of biomaterial had studied and applied in bridging repairing model of rat sciatic nerve such as the chitosan [6], the polylactic acid [7], the polyglycolic acid [8], the pro-anthocyanidin cross-linked gelatin [9], and the genipin cross-linked gelatin [4, 10]. However, the previous study with success nerve regeneration was used a small gap of 10 mm or less [11]. The current study assessing the large gap repair of nerve of more than 15 mm in rat sciatic nerve regeneration cuffing model is necessary to fully reveal the benefits of using bridging tubes, since the inherent regenerative capacity of the peripheral nervous system in animals is quite efficient over short gaps. There were several growth-promoting factors that affect regeneration have been explored, such as the nerve growth factor [12], brain-derived neurotrophic factor [13], glial cell line-derived neurotrophic factor [14], and insulin-like growth factors [15]. Recently, the immune system has been demonstrated to have important functions during nerve regeneration [16], e.g., macrophages are abundant at the site of injury and are involved in removing debris and secreting growth factors to promote nerve regeneration [17]. The growth-promoting role of macrophages in injured nerves might be explained by their phenotype [18]. Macrophages with the pro-healing (M2) phenotype were shown to support tissue repair by producing anti-inflammatory cytokines [19], also like interleukin (IL)-6 [20] or Janus kinase 2 (JAK2) inhibitor [21]. On the contrary, macrophages with the pro-inflammatory (M1) phenotype were detrimental to neuronal growth as they created local inflammatory conditions with high levels of oxidative metabolites and pro-inflammatory cytokines [22]. Therefore, it is an important issue to understand the contribution of different cytokines in nerve regeneration associated with macrophages infiltration.

In this research, a silicone rubber nerve guide filled with collagen gels containing cytokine interferon (IFN)-γ or IL-4 was used to establish a nerve bridge across a 15-mm gap in rat sciatic nerves. In addition, nerve growth factor (NGF) and saline mixed with collagen filled in the bridging guide were considered as the positive and negative controls, respectively. The therapeutic bulk effects of these fillings were then evaluated by the examination of the electrophysiological nerve function, expression of calcitonin gene-related peptide (CGRP) in the spinal cord, and recruitment of macrophages to nervous tissues; retrograde labeling of dorsal root ganglions (DRGs) with fluorogold; and morphometric observations of regenerated nerves in the bridging chamber. Finally, we studied changes in the mRNA levels of fibroblast growth factors (FGF), NGF, transforming growth factor (TGF)-β, IL-1, platelet-derived growth factor (PDGF), and IFN-γ in the regenerated rat sciatic nerve segments.

## Results

### Nerve regeneration success rates

Firstly, we noted that the gross examination of the silicone rubber chambers at 6 weeks revealed much higher rates of successful regeneration in the NGF and IL-4 groups (Table 1), at 100% (10 of 10, *p* = 0.0054) and 80% (8 of 10, *p* = 0.0849) compared to the saline group (40%, 4 of 10). However, only 30% (3 of 10, *p* = 0.8251) in the IFN-γ group exhibited such a regenerated nerve across the 15-mm gap.

**Table 1:** Success rates of regenerated nerves across the 15 mm gaps

Groups	Biological agent included in the silicone rubber tube			
	PBS	NGF^a^	IL-4^b^	IFN-γ
Success rate (total numbers)	4 (10)	10 (10)	8 (10)	3 (10)
*p* value^c^	-	0.0054	0.0849	0.8251

^a^No myelinated axons were seen in three rats though their nerve gaps had been reconnected

^b^No myelinated axons were seen in one rat though its nerve gap had been reconnected

^c^All *p* values were estimated by Fisher’s exact test and compared to the PBS group

### Electrophysiological measurements

**Fig. 1:**
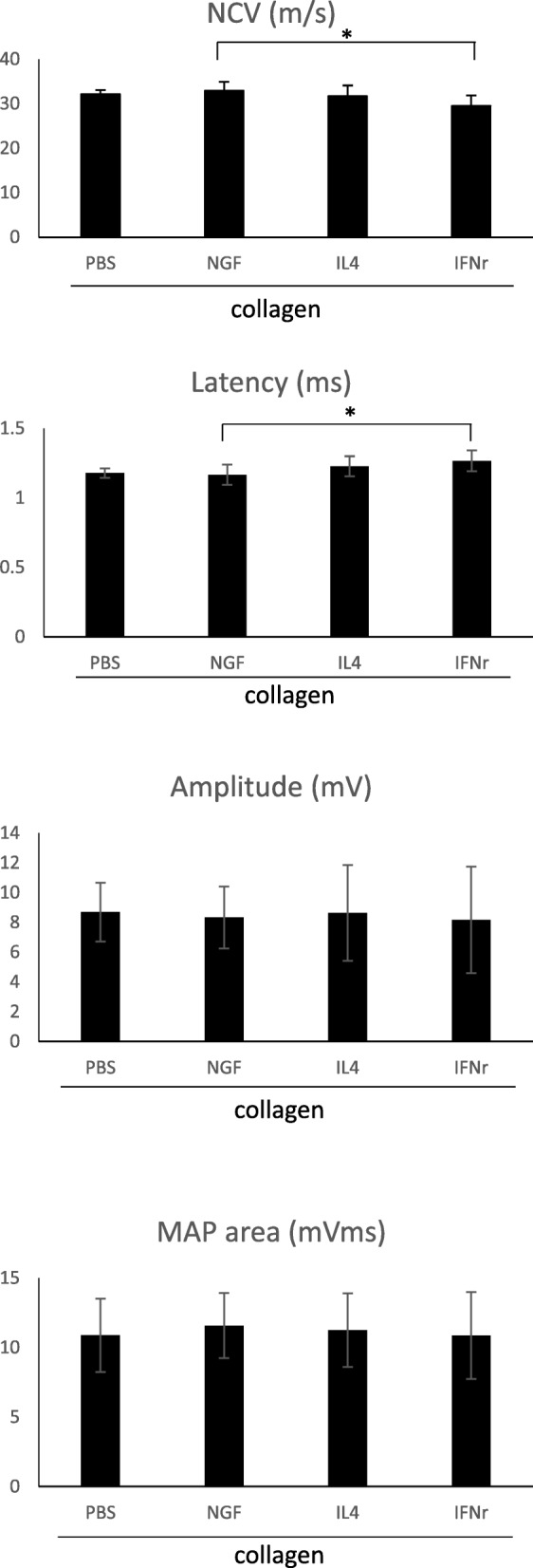
Analysis of evoked MAPs, including NCV, latency, peak amplitude, and area under the MAP curves. *Significant differences between conditions, *P* < 0.05

**Fig. 2:**
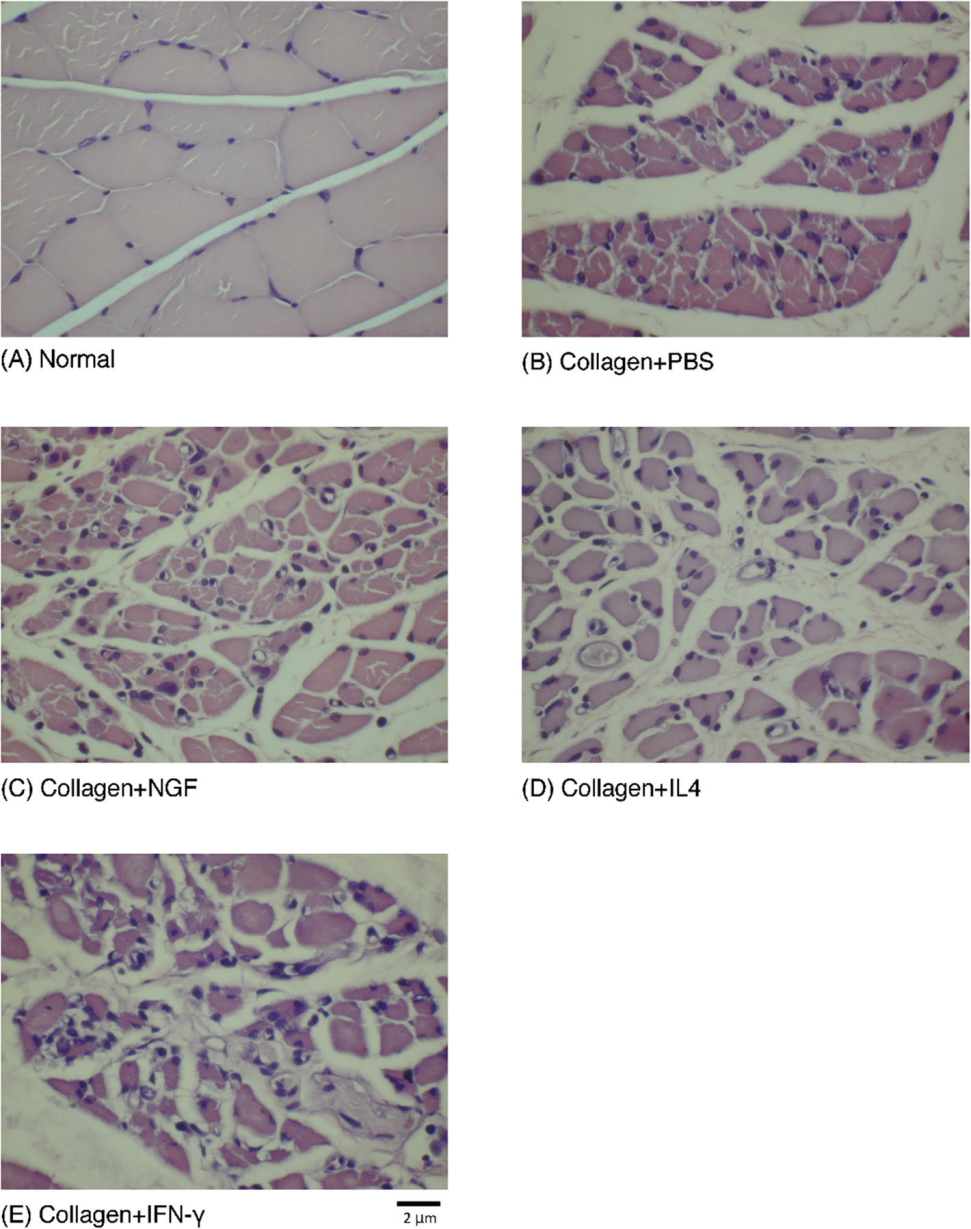
**a** Cross section of the muscle bundle shows closely packed fibers in normal rat gastrocnemius muscle. **b-e** Evident muscle atrophy is noted with increased fibrosis and fatty infiltration in groups repaired using silicone rubber conduits with different fillings. Scale bar = 2 μm

Significant differences were seen in the neurophysiological changes in the increasing NCV and the reducing latency of NGF group, compared to IFN-γ group (Fig. 1a and b). No significant differences were noted in the other neurophysiological measurements, including amplitude and MAP area (Fig. 1c and d) that could be caused by serious atrophy of the gastrocnemius muscle even after 6 weeks of recovery (Fig. 2)

### Retrograde labeling with fluorogold

**Fig. 3:**
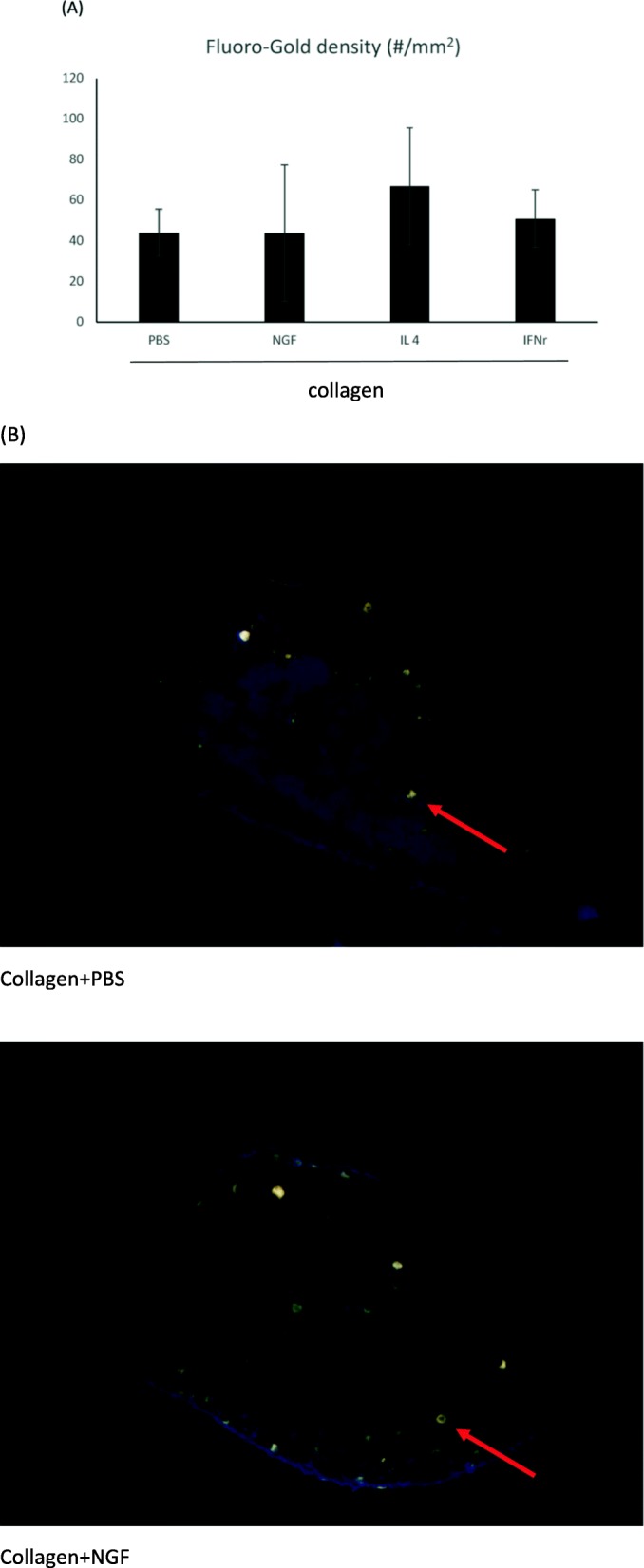
**a** No significant difference was seen in the number of fluorogold-labelled cell in DRGs (arrows) among the four groups with different conduit fillings. **b** Representative images of the retrograde axonal tracing with fluorogold. Scale bar = 250 μm

Morphometric data among the four groups didn’t reach the significant level which could be caused by large standard deviations in the regenerating nerves (Fig. 3a). However, the fluorogold-labeled cells in the cryostat section revealed that the migrating axons had overcome the nerve gap in the bridging tube and reached the DRG, indicating successful neuronal connectivity (Fig. 3b-e).

### CGRP-IR in the dorsal horn following injury

**Fig. 4:**
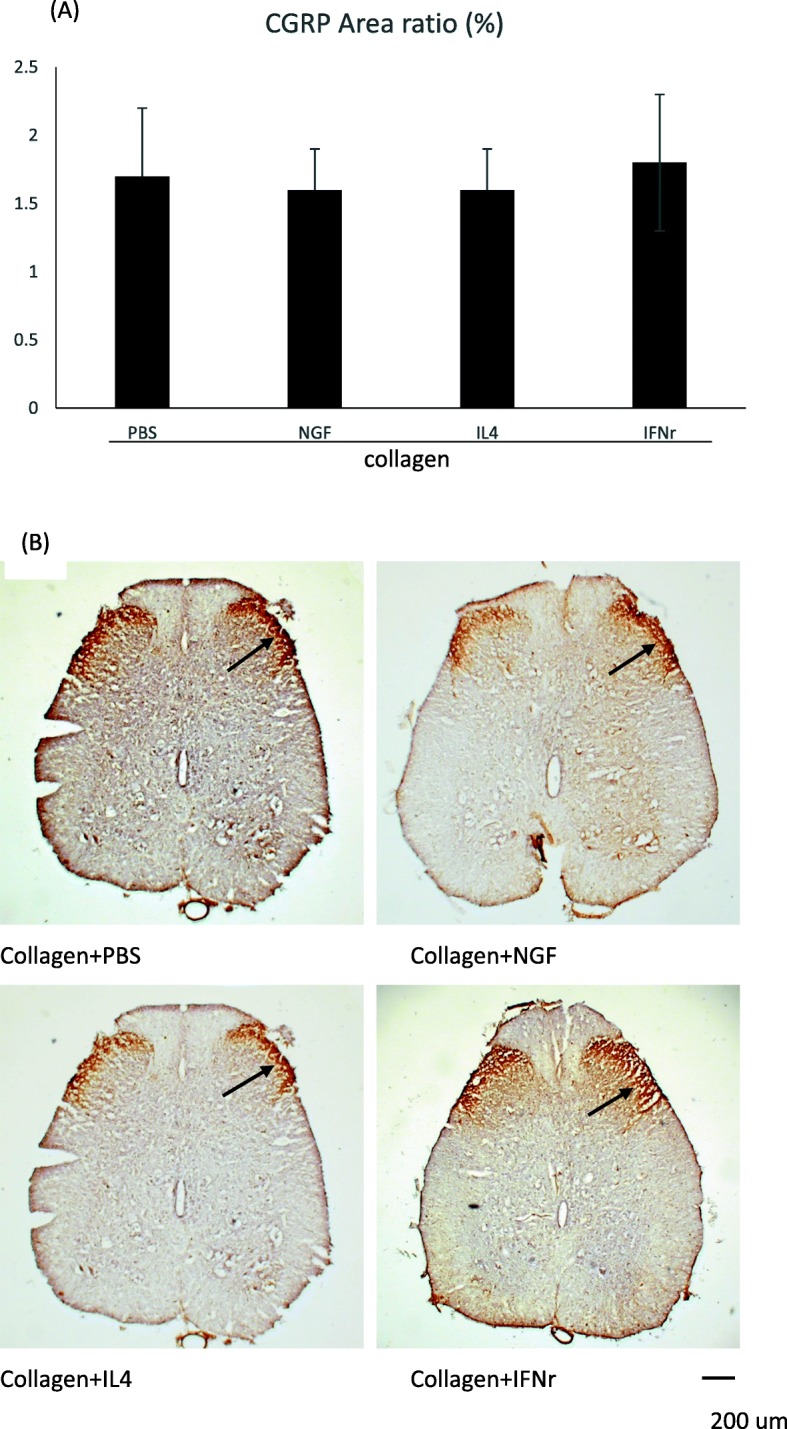
Photomicrographs of CGRP-IR in dorsal horn in the lumbar spinal cord after injury. **a** The positive CGRP-IR area ratio, **b** Cross-sectional histological view of CGRP-labeled fibers (arrows) via immunohistochemical staining. Scale bar = 200 μm

Immunohistochemical staining showed that the CGRP-labeled fibers were observed in the lamina I-II regions of the dorsal horn ipsilateral to the injury in all the rats (Fig. 4). However, it was noted that the proportions of the area occupied by CGRP-immunoreactive cells in the four groups were not significantly different.

### Macrophages recruited in the distal nerve ends

**Fig. 5:**
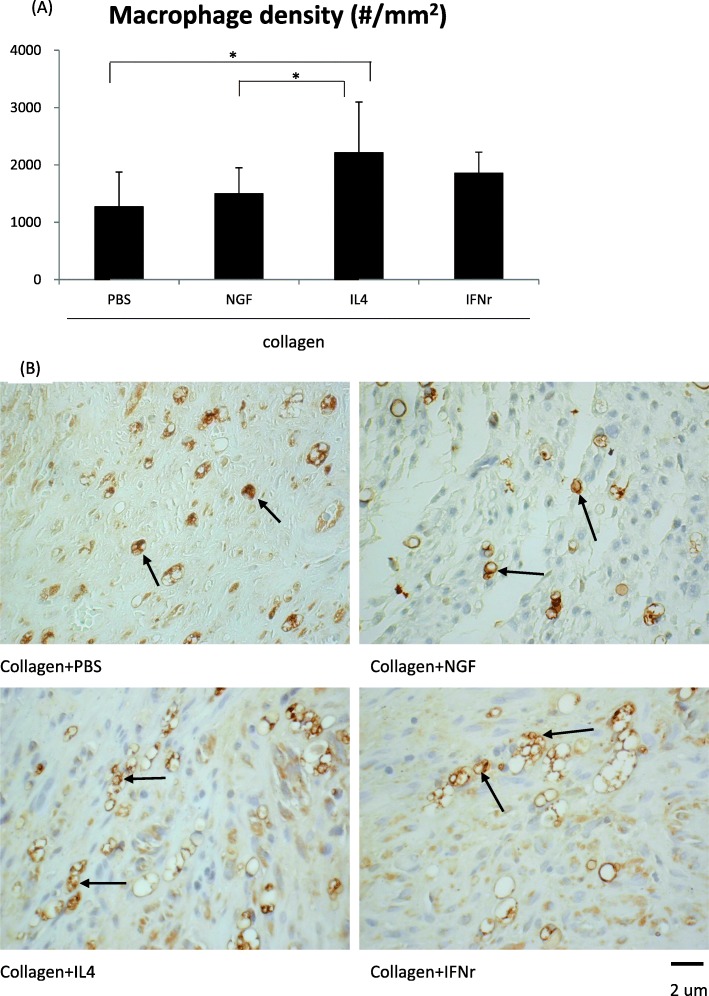
Macrophages stained with CD68 and Iba-1 immunoreactivity. **a** The macrophage density, **b** Representative photographs of stained macrophages (arrows) in regenerated nerves treated with different conduit fillings. *Significant differences between conditions, *P* < 0.05. Scale bar = 2 μm

It was noted that a significantly higher density of macrophages (CD68^+^ phagocytotic cells) was recruited into the regenerated sciatic fascicles in the IL-4-treated rats, compared to the saline controls and the NGF-treated animals (Fig. 5, *p* < 0.05). This result indicated that deliberate superimposition of macrophage-related cytokine could dramatically enhance influx of macrophages in injured nerves.

### Sciatic nerve regeneration

**Fig. 6:**
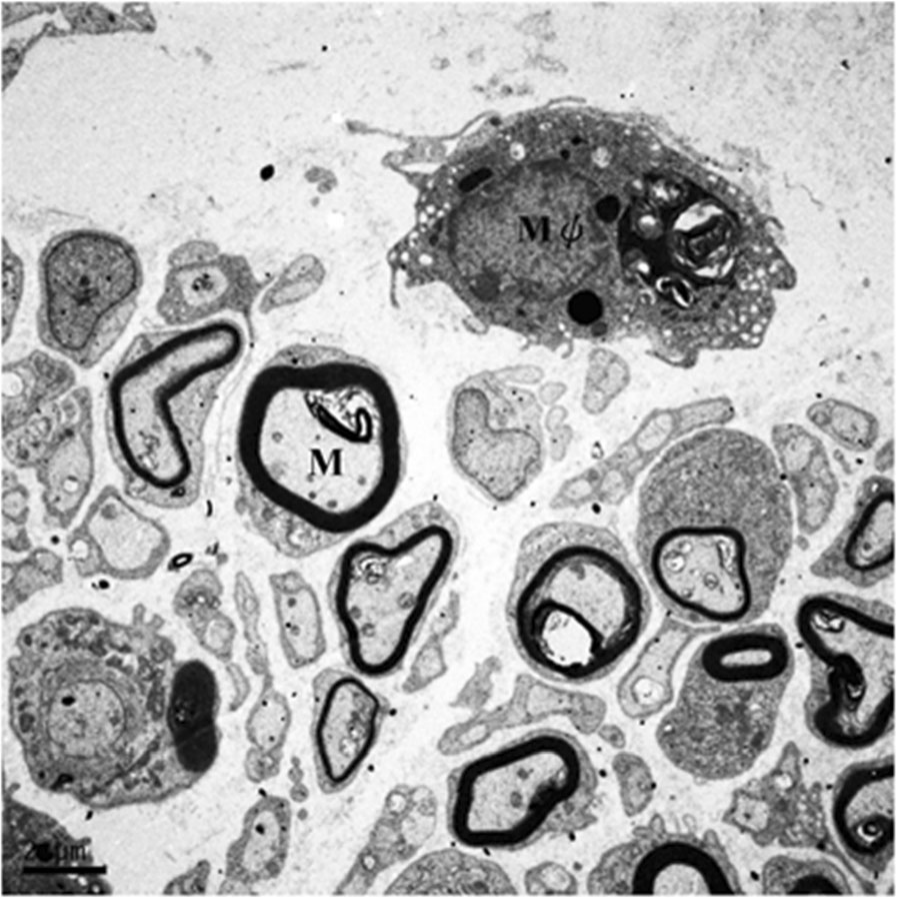
Ultrastructure image of regenerated sciatic nerve by TEM to show the macrophage (Mφ) and the myelinated axon (M). Scale bar = 2 μm

**Fig. 7:**
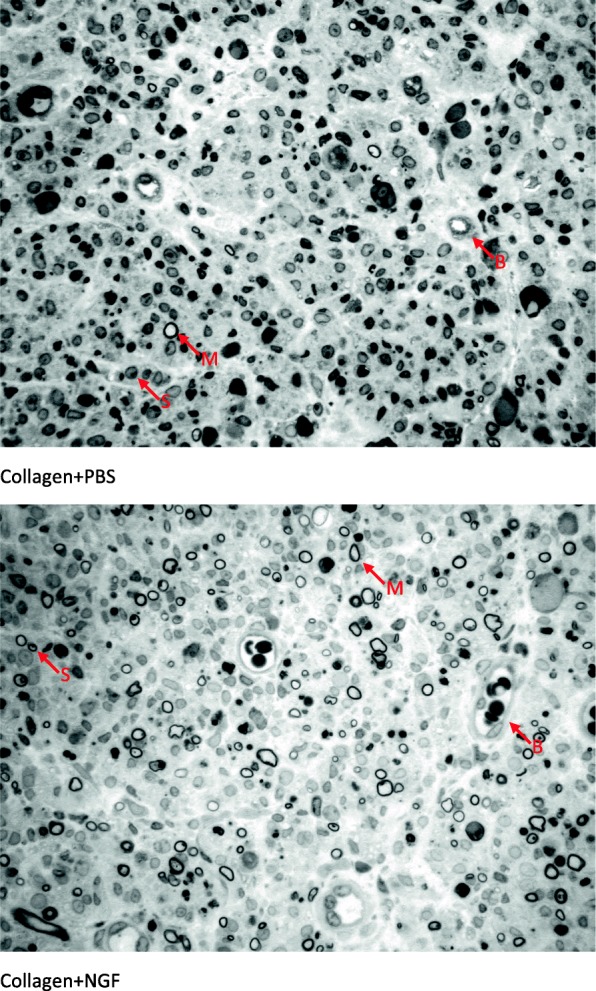
Light micrographs of representative cross sections in regenerated nerves treated with different conduit fillings. Myelin sheath (M) of the regenerated axon was stained dark blue with the toluidine blue. Blood vessels (B) and Schwann cells (S) are interspersed among these axons. Scale bar = 20 μm

**Fig. 8:**
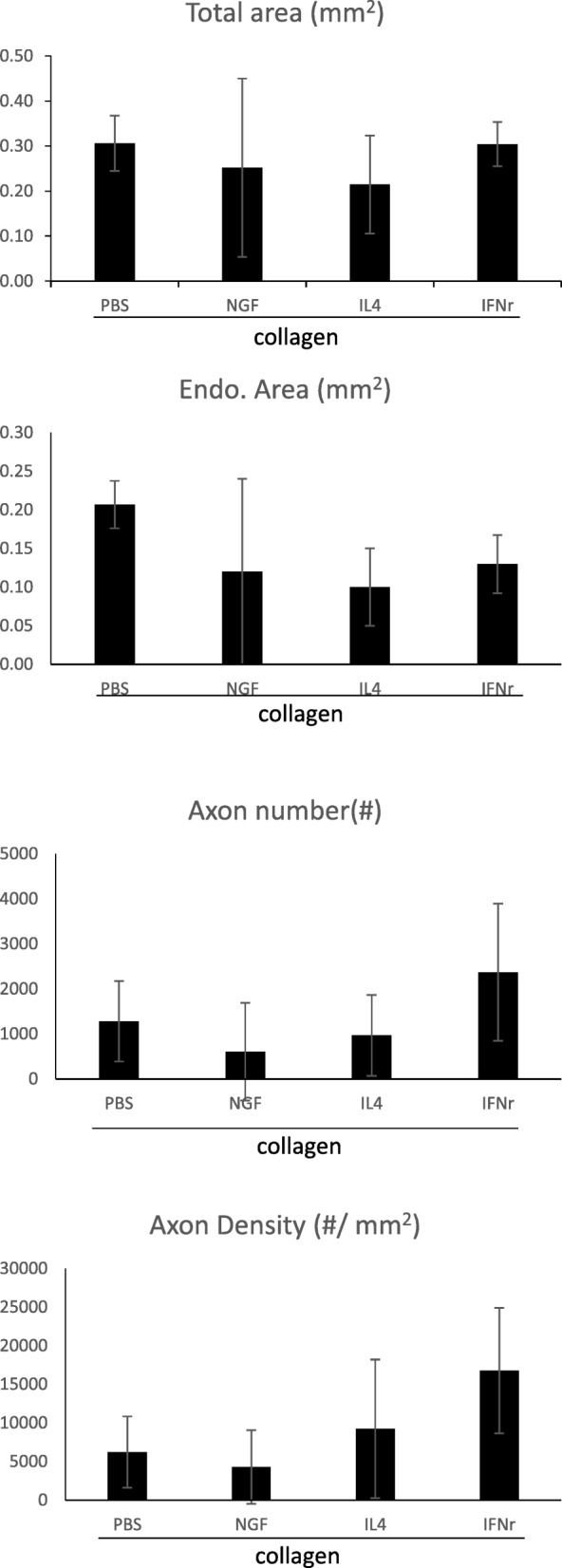
Morphometric comparisons of regenerated nerves, including total area (mm^2^), endoneurial area (mm^2^), axon number (#), and axon density (#/mm^2^)

Figure 6 shows an ultrastructural assay of representative regenerated nerves conducted using TEM. A relatively large fraction of the core in the regenerated nerve contained collagenous endoneurial connective tissue with macrophages which were proximity to Schwann cells with myelinated axons, indicating stimulation of inflammation is a pivotal factor to induce a regenerative response in axonal regeneration models. Figure 7 shows the representative cross-sections of the regenerated nerve specimen. As shown in the TEM photo, most of the images of regenerated nerves obtained from light microscopy displayed a similar ultrastructural organization among the four groups. The Schwann cells and blood vessels were interspersed among myelinated axons. However, we noted that three of the 10 regenerated nerves in the NGF group had an amorphous core which was wholly composed of a fibrin matrix, in which only blood vessels and Schwann cells were observed. Similarly, one of the eight regenerated nerves in the IL-4 group had such an immature ultrastructural organization. We noted that large deviations were seen in the regenerating nerve indicators, such as total nerve area, endoneurial area, axon number, and axon density (Table 2), resulting in no significant differences in the comparisons of these indicators among the four groups (Fig. 8).

**Table 2:** Indicators of regenerating nerves

	Total area (mm^2^)	Endoneurial area (mm^2^)	No. of axons (#)	Density (#/ mm^2^)
Group PBS				
	0.33	0.24	1140	4809.1
	0.30	0.20	2532	12,972.1
	0.22	0.17	431	2546.7
	0.37	0.23	1010	4483.8
Mean	0.31	0.21	1279	6203.0
SD	0.06	0.03	891	1622.0
Group NGF				
	0.21	0.04	22	569.2
	0.20	0.02	149	6565.2
	0.50	0.30	3426	11,328.3
	0.10	0.06	15	149.9
	0.42	0.20	1097	5352.6
	0.10	0.03	233	7038.8
	0.21	0.09	1118	11,778.6
	0.08	0.05		
	0.63	0.33		
	0.06	0.04		
Mean	0.25	0.12	866	6111.8
SD	0.20	0.12	1226	4604.9
Group IL-4				
	0.37	0.18	2580	14,366.2
	0.12	0.06	51	800.6
	0.22	0.06	1372	23,704.1
	0.20	0.09	1293	14,044.7
	0.37	0.13	560	4164.2
	0.17	0.12	285	613.2
	0.20	0.10	1581	16,126.8
	0.06	0.02		
Mean	0.21	0.10	1103	10,545.7
SD	0.11	0.05	874	8807.9
Group IFN-γ				
	0.36	0.15	2777	8548.1
	0.29	0.15	3646	23,858.7
	0.26	0.09	681	7890.9
Mean	0.30	0.13	2368	16,765.9
SD	0.05	0.04	1524	8131.7

**Fig. 9:**
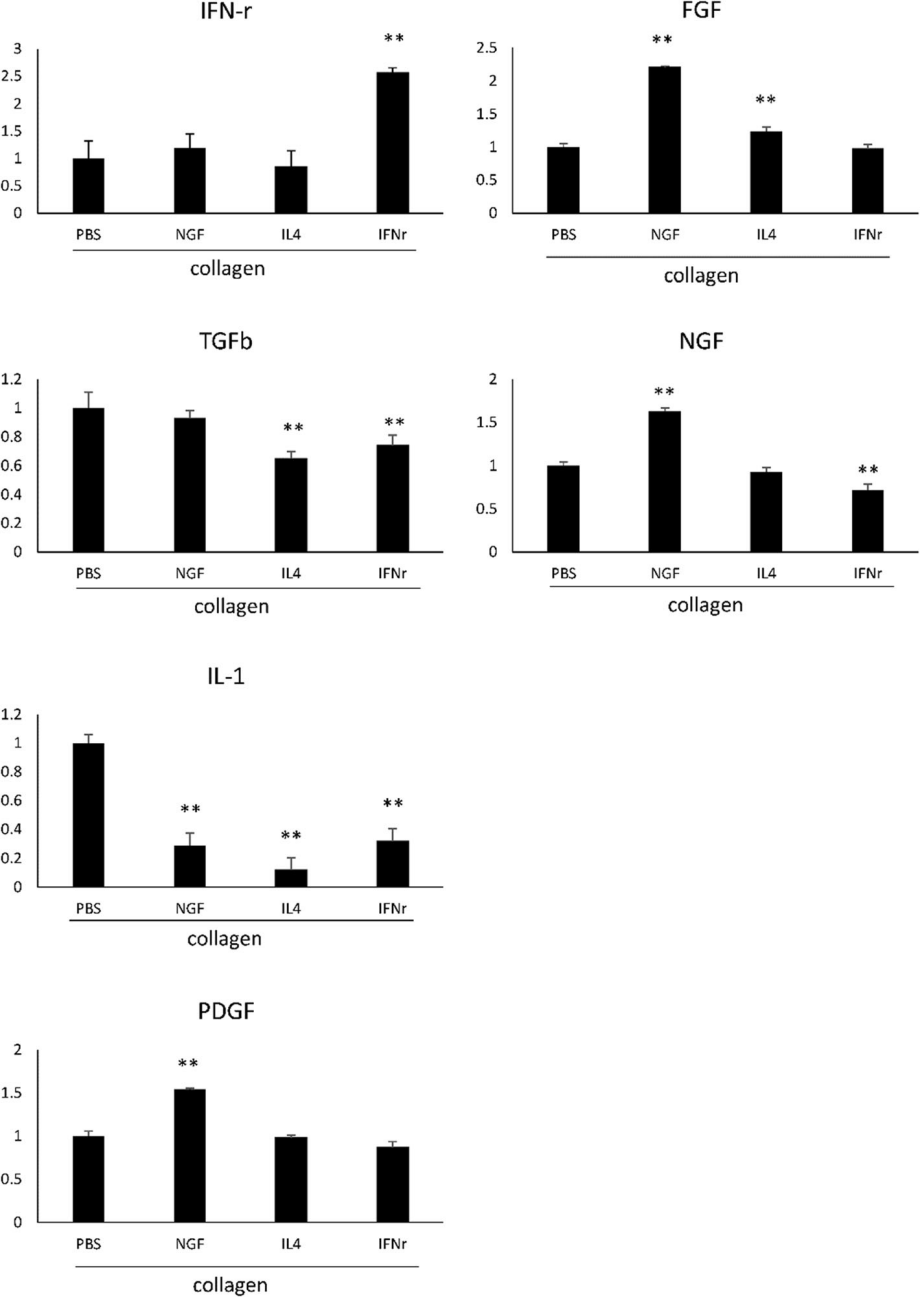
Cytokine expression changes in regenerated nerves estimated by ELISA for IFN-γ, IL-1, NGF, FGF, PDGF, and TGF-ß

**Fig. 10:**
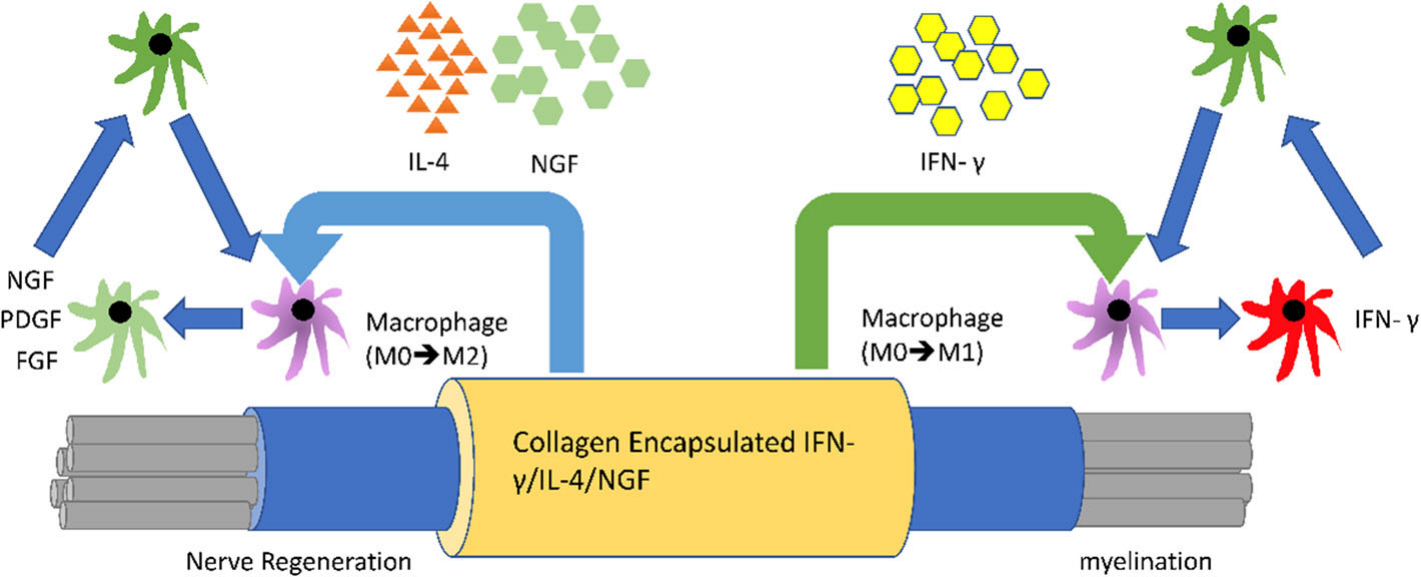
Schematic description of the micro-environmental regulation of different guide fillings for nerve regeneration

Figure 9 demonstrates that micro-environments among these different guide fillings for nerve regeneration should be inducible, such as the paracrine cytokines expressed around the injured tissue. The secreted cytokines, like IL-1 and NGF, were suppressed by the IFN-γ, and the paracrine FGF, NGF, and PDGF were elevated by NGF induction through the surrounding tissue. Interestingly, the IL-1 expression changes were significantly reduced in all three treatments, including NGF, IL-4, and IFN-γ. The microenvironmental changes of large gap nerve regeneration clued the pivotal changes of immune mediators in these different guide fillings for nerve regeneration as shown in Fig. 10. The IL-1, which is macrophage-derived inflammatory cytokine, should be associated with the modulation of macrophage cell infiltration, inflammation and tissue repair at injured sites.

## Discussion

Most of previous studies with the rat nerve regeneration model commonly used a nerve gap less than 10 mm long [23]. However, the natural regeneration ability of rat peripheral nerve is quite strong. If the nerve gap designed in the experiment is too short, it may not be able to reveal the benefits of microenvironment changes in the bridging conduit. Moreover, we know that the macrophages could modulate regeneration in the nervous system, but it is unclear if they support or hinder regenerative biophysical cascades after injury. In the present study, we therefore repaired dissected rat sciatic nerves with 15 mm apart using silicone rubber conduits filled a mixture of collagen and different cytokines. As a result, both the NGF and IL-4 guide fillings could significantly accelerate the formation of nerve tissues re-connecting the injured stumps, showing their potential growth-promoting capability for peripheral nerve regeneration. The NGF has long been identified involving in the regulation of growth and survival of regenerating neurons [24]. It may bind with tropomyosine receptor kinase A or low-affinity NGF receptor to reduce neuronal degeneration [25, 26], accelerate peripheral nerve regeneration [27, 28], and suppress inflammation [29, 30]. Conversely, the IFN-γ was found that it may hinder the regenerative processes. We believe that local inflammatory conditions induced by the filled cytokines may contribute to these events. These results also agree with previous findings that more than the extent of macrophage presence, their specific phenotype at the site of injury could also influence the regenerative outcomes [18, 31].

As we know, successful nerve regeneration relies on both injured axons and non-neuronal cells such as immune cells, especially the macrophages [32]. Macrophages, in addition to being able to rapidly remove myelin debris from degenerating axons during nerve growth, can also secrete nerve-growth factors to assist in regenerative processes [33, 34]. When the nerve is injured, macrophages will accumulate in the injured area to participate in the Wallerian degeneration. They also can be polarized to M2 macrophages to support nerve repair by producing anti-inflammatory cytokines [35–38]. In the present study, macrophage infiltration density was significantly increased in the distal sciatic nerve after injury in the IL-4 group. This may cause an advance Wallerian degeneration accompanied with more secretion of neurotrophic factors, resulting in enhanced nerve regeneration. Similar results have been reported that the IL-4 may activate M2 macrophages to produce anti-inflammatory cytokines which could mediate angiogenesis, cell replacement and matrix remodeling while suppressing destructive immunity [39]. The activation of M2 macrophages may promote Schwann cell infiltration and accelerate axonal growth in regenerating nerves. On the contrary, the IFN-γ could activate M1 macrophages which may produce high levels of oxidative metabolites and pro-inflammatory cytokines which not only have adverse effect on regenerating axons, but also hinder the regenerative process [39].

Our study possibly existed several limitations as followings. First, the constraint of sample size of animals may limit our observations in this in vivo test, though the quality of nerve regeneration and the clustering of morphometric data still show that NGF and IL-4 groups were better with higher rates of successful regeneration with improved NCV and latency as compared to those in the groups of IFN-γ and saline. However, electrophysiological evaluations of amplitude and MAP area of the regenerated nerves failed to show such convincing evidences. This could be caused by misdirected connections of regenerating nerve fibers and the serious muscle atrophy during the regenerative periods [40]. Second, the NGF- and IL-4 induced cytokine expression changes were demonstrated that the significant reduction of IL-1, which might be attributable for late-phase would be present lower mRNA level changes after sciatic nerve injury [41], and the reduction of IL-1 level should be associated with its feedback control, like antagonist (IL-1RA) could suffer to be destructive to peripheral nerve attempting to repair itself [42]. Thus, the IL-4 stimulus nerve regeneration should be associated with the scarring formation with more macrophage or fibroblast infiltration as one of the possible inferences. Third, the injury-related signals derived from regenerating sciatic nerves and retrogradely transported to neurons in the dorsal horn were so strong that the CGRP expression could not be discerned among the four groups with different modifications in the bridging chambers.

In conclusion, our study demonstrates that NGF and IL-4 show potential growth-promoting capability for peripheral nerve regeneration. On the contrary, IFN-γ may impede the regeneration processes. The modulation of macrophage phenotype at the site of peripheral nerve injury may regulate the regenerative potential after injury that has significant implications for the treatment of long peripheral nerve gaps.

## Methods

### Surgical preparation of animals

Forty adult Sprague-Dawley rats underwent the placement of silicone chambers. The animals were anesthetized with an inhalational anesthetic technique (Aerrane®, Baxter, USA). Following the skin incision, the fascia and muscle groups were separated using blunt dissection, and the right sciatic nerve was severed into proximal and distal segments. The proximal stump was then secured with a single 9–0 nylon suture through the epineurium and the outer wall of the silicone rubber chamber (1.47 mm ID, 1.96 mm OD; Helix Medical, Inc., Carpinteria, CA, USA). The animals were divided into four groups A-D representing the PBS, NGF, IL-4, and IFN-γ treatments, respectively. In group A (*n* = 10), the chambers were filled with a mixture of 2.4 mg/mL collagen (Vitrogen®, Cohesion, Palo Alto, CA, USA) and normal saline at a 1:1 volumetric addition. In group B (*n* = 10), a mixture of the Vitrogen® collagen (2.4 mg/mL) and 50 ng/mL nerve growth factor (NGF) (S0513, Sigma-Aldrich, Inc., St. Louis, MO, USA) at a 1:1 volumetric ratio was filled in the chambers. Similarly, the chambers in groups C (*n* = 10) and D (*n* = 10) were filled with a mixture of the Vitrogen® collagen (2.4 mg/mL), and 1 μg/mL IL-4 (400–04, PeproTech, Inc., NJ, USA) or 1 μg/mL IFN-γ (400–20, PeproTech, Inc., NJ, USA) at a 1:1 volumetric addition, respectively. Collagen filled in the chambers was to prevent these loadings from leakage.

The volume of the chamber lumen was approximately 25.5 μL. These fillings, which were in the liquid state, were injected through a pre-cooled micropipette into the lumens by passing the tip of the needle inside the silicone rubber chambers, and the loading was done as slow as possible to prevent the formation of air bubbles. The mixture polymerized into a gel at 37 °C, the animal body temperature. The distal stump was then secured into the other end of the chamber. Both the proximal and distal stumps were secured at a depth of 1 mm in the chamber, leaving a 15-mm gap between the stumps. The muscle layer was re-approximated with 4–0 chromic gut sutures, and the skin was closed with 2–0 silk sutures. All the animals were housed in a temperature (22 °C)- and humidity (45%)-controlled room with 12-h light cycles, and they had access to food and water ad libitum. All chambers remained in place for 6 weeks, at which time the nerves were re-exposed, and the chambers examined for the presence of regenerated nerve across the 15-mm gap. Each rat was maintained at a lower oxygen flow rate (0.8 to 1.5 L/min) of isoflurane anesthesia (3 to 5%) and placed on a stainless-steel tray. The rodents were sacrificed by inhalant anesthetic overdose in a plastic bell jar, followed by bilateral thoracotomy. All the animal experiments conformed to the Animal Protection Act, Taiwan, and the Institutional Animal Care and Use Committee (IACUC) of China Medical University, Taichung, Taiwan (No. 102–45-N) for the care and use of animals.

### Electrophysiological analysis

Sciatic nerves were exposed in the anesthetized animals for electrophysiological testing. The stimulating cathode composed of a stainless-steel monopolar needle was placed at the sciatic nerve trunk 5 mm proximal to the transection site and the anode was placed 3 mm proximally to the cathode. Conductive nerve velocity (NCV), amplitude, latency, and evoked muscle action potentials (MAPs) of the gastrocnemius muscles were then recorded using a computer system from BIOPAC Systems, Inc. (Goleta, CA, USA). The time for the electrical impulse to travel from the stimulation to the recording site in the gastrocnemius muscles was measured as the latency. The areas from baseline to the maximal negative peak and amplitudes were calculated. The NCV was then obtained by dividing the distance between the stimulating sites at the sciatic nerve proximal and distal to the bridging conduit by the difference in the latency time.

### Retrograde labeling with fluorogold

After electrophysiological recording, a 2% fluorogold suspension was prepared by dissolving fluorogold in distilled water, stored at 4 °C in the dark, and directly injected using a Hamilton micro-syringe into the common peroneal nerve and posterior tibial nerve. Five days later, the rats were trans-cranially perfused sequentially with 200 mL of 0.9% saline, followed by cold 4% paraformaldehyde in 0.1 M PBS. Next, L4 and L5 DRGs on the same side of the injury were dissected and soaked in 4% paraformaldehyde for post-fixation overnight, and in 30% phosphate-buffered sucrose solution for additional overnight. DRGs of 40-μm thickness were then obtained from longitudinal sections of the spinal cord. After drying, the section was mounted and observed under an ultraviolet fluorescence microscope (Olympus Ckx41 Culture Microscope).

### Histological processing

The animals were perfused trans-cranially and their L4 spinal cord was removed and post-fixed in the same abovementioned fixative material for 4 h. All the specimens were immersed in 30% sucrose at 48 °C overnight, embedded in the cutting temperature solution, frozen at − 20 °C, and sliced into 18-mm slices fixed on poly-L-lysine coated slides. In order to inactivate all endogenous peroxidase activity in the specimens, they were incubated in 0.3% H_2_O_2_ and immersed in Protein Block solution (RE7102; Novocastra Laboratories Ltd., Newcastle upon Tyne, UK) to inhibit all non-specific binding sites. The specimens were then treated serially with anti-CGRP antibody 1:1000 (Calbiochem, San Diego, CA, USA), Post Primary Block (RE7111; Novocastra Laboratories Ltd., Newcastle upon Tyne, UK), and secondary antibody (Novolink Polymer RE7112). The diaminobenzidine solution was used to develop the L4 spinal cord sections and hematoxylin for counterstaining. The middle regions of the regenerated nerves were removed and fixed, and treated with 0.5% osmium tetroxide before dehydration and embedding in Spurr’s resin. A Leica EM UC6 microtome was used for alignment with diamond knives (Leica Biosystems, Wetzlar, Germany), and 2-μm-thick sections were cut. After staining with toluidine blue, the specimen were observed under an optical microscope (Olympus IX70 Fluorescence Microscope). Also, 70-nm ultra-thin sections were cut, stained with uranyl acetate and lead citrate, and observed by transmission electron microscopy (TEM) at 100 kV (Leica, Wetzlar, Lahn-Dill-Kreis, Germany).

Immunofluorescence staining was used to observe the macrophages (CD68+) in the distal regions of the nerve stump. The specimens were treated with 10% bovine serum albumin containing 0.4% Triton X-100 for 1 h, followed by 4 °C overnight treatment with primary antibodies in blocking solution. The primary antibodies were anti-CD68 (1:200; Serotec, Hercules, CA, USA) and anti-Iba1 (1:100, Bioss, Woburn, MA, USA). Tissue sections were rinsed and immersed in secondary antibodies (Alexa Fluor 488 or 594; Abcam, Cambridge, UK) for 1 h at room temperature. The images were then acquired with an SP2/SP8X confocal microscope (Leica, Wetzlar, Lahn-Dill-Kreis, Germany).

### Image analysis

To observe the tissue specimens, an image analyzer system (Image-Pro Lite, Media Cybernetics, Rockville, MD, USA) coupled to a light microscope was used. The CGRP-immunoreactivity in the dorsal horn of the lumbar spinal cord was considered positive if it was 5-fold denser than the background levels. The ratio of positive CGRP-immunoreactive areas in the dorsal horn ipsilateral to the injury and the number of neural components in each nerve section were measured. At least 50% of the sciatic nerve section was randomly chosen from each specimen to estimate the number of myelinated axons and the macrophages as well as the nerve areas.

### Enzyme-linked immunosorbent assay (ELISA)

Production of IFN-γ, IL-1, NGF, FGF, PDGF, and TGF-ß was quantified using ELISA kits (Abcam, Cambridge, UK) following the manufacturer’s instructions. Briefly, total blood (10 mL) was collected from the exposed heart at the time of decapitation of deeply anesthetized animals and incubated at room temperature for 30 min. Next, the serum was obtained by centrifuging whole blood at 2000 rpm at 4 °C for 15 min and analyzed using an ELISA kit. The concentrations of IFN-γ, IL-1, NGF, FGF, PDGF, and TGF-ß were measured through correlation with a standard curve. Blank disks were used as control.

### Statistical analyses

All data were collected by the same observer, and raw data for the electrophysiological and histological experiments are shown in Table 2. The statistical analyses of the continuous variables which expressed as the mean ± standard deviation (SD), and category variables or groups comparison of proportion were using Chi-Square (χ2) analysis of variance from SAS Enterprise Guide 7.1/JMP 14 pro (SAS Institute, Inc., Cary, NC, USA). All statistically significance level was set at *P* < 0.05.

## Data Availability

All data generated or analysed during this study are included in this published article.

## Abbreviations

CGRP: Calcitonin gene-related peptide
ELISA: Enzyme-linked immunosorbent assay
FGF: Fibroblast growth factors
IFN-γ: Interferon-γ
IL-4: Interleukin-4
MAPs: Muscle action potentials
NCV: Conductive nerve velocity
NGF: Nerve growth factor
PDGF: Platelet-derived growth factor
TGF- β: Transforming growth factor-β
